# A Rapid, Highly Efficient and Economical Method of *Agrobacterium*-Mediated *In planta* Transient Transformation in Living Onion Epidermis

**DOI:** 10.1371/journal.pone.0083556

**Published:** 2014-01-08

**Authors:** Kedong Xu, Xiaohui Huang, Manman Wu, Yan Wang, Yunxia Chang, Kun Liu, Ju Zhang, Yi Zhang, Fuli Zhang, Liming Yi, Tingting Li, Ruiyue Wang, Guangxuan Tan, Chengwei Li

**Affiliations:** 1 Key Laboratory of Plant Genetics and Molecular Breeding, Zhoukou Normal University, Zhoukou, People's Republic of China; 2 Department of Life Science, Zhoukou Normal University, Zhoukou, People's Republic of China; 3 College of Life Science, Henan Agricultural University, Zhengzhou, People's Republic of China; University of Louisville, United States of America

## Abstract

Transient transformation is simpler, more efficient and economical in analyzing protein subcellular localization than stable transformation. Fluorescent fusion proteins were often used in transient transformation to follow the *in vivo* behavior of proteins. Onion epidermis, which has large, living and transparent cells in a monolayer, is suitable to visualize fluorescent fusion proteins. The often used transient transformation methods included particle bombardment, protoplast transfection and *Agrobacterium*-mediated transformation. Particle bombardment in onion epidermis was successfully established, however, it was expensive, biolistic equipment dependent and with low transformation efficiency. We developed a highly efficient *in planta* transient transformation method in onion epidermis by using a special agroinfiltration method, which could be fulfilled within 5 days from the pretreatment of onion bulb to the best time-point for analyzing gene expression. The transformation conditions were optimized to achieve 43.87% transformation efficiency in living onion epidermis. The developed method has advantages in cost, time-consuming, equipment dependency and transformation efficiency in contrast with those methods of particle bombardment in onion epidermal cells, protoplast transfection and *Agrobacterium*-mediated transient transformation in leaf epidermal cells of other plants. It will facilitate the analysis of protein subcellular localization on a large scale.

## Introduction

Onion (*Allium cepa* L.), one kind of biennial herb Liliaceae plant, has been used as classical experimental materials in analyzing structure of plant cells, distribution location of DNA and RNA, reducing sugar of plant tissues [1], plasmolysis and recovery of plant cells [2], [3], karyotype [4], protein subcellular localization and interaction [5]–[7].

Imaging subcellular localization of proteins in living cells has become an important tool for defining protein function. Fluorescent fusion proteins are ideal marker non-enzymatic protein systems for imaging protein subcellular localization in living cells, which have many apparent advantages, such as stable fluorescence properties, easy observation, visualization in living cells, non-toxic to cells, non-specifity for species, without interference to false positives and no substrate etc. In addition, expression of fluorescent fusion proteins had also been used to investigate protein interaction, trafficking, turnover, movement and inheritance in living cells [8].

Transient transformation assays, which were conducted by using particle bombardment [9], [10], protoplast transfection [11], and *Agrobacterium*-infiltration [12], [13], [14], had been used to analyze gene function and protein-protein interactions in *Arabidopsis* [15], [16] and rice [11], [17], hybrid aspen [18], maize [19], [20], potato [21], [22], soybean [23]–[25], tomato [26]–[28], wheat [23], [29], [30], white spruce [31], celery and carrot [32].

However, most transient transformation methods have certain disadvantages, such as the lower transformation efficiency, equipment dependency and auxiliary material needed for particle bombardment, complex preparation procedures required for protoplasts transfection. In addition, for transient transformation in plants having complex outline of epidermal cells and the bushy epidermal hairs, laser scanning confocal microscope was usually needed to get ideal micro-images, which increased the reliability on expensive equipments. To avoid these disadvantages, we developed an *in planta* transient transformation method in living onion epidermal cells by using *Agrobacterium*-mediated infiltration. In this protocol, infiltration liquid of *Agrobacterium* carrying constructed vectors were injected into the interface between adaxial epidermis and mesophyll of onion bulb scales, which played an important role in yielding high transformation efficiency, and kept in the living onion bulb for about three days. With this simple method a higher frequency of transformation was achieved without expensive equipments in comparison with other transient transformation methods like protoplast transfection of *Spinacia oleracea* [33], *A. thaliana* [34] and *Populus euphratica* [35], agroinfiltration of *Arabidopsis* epidermal cells and particle bombardment of onion cells *in vitro*. In addition, following this method it took about 3 days for agroinfiltration to get ideal transient transformation efficiency in living onion epidermis. Therefore, the developed method was rapid, highly efficient, equipment independent and low-cost, which will benefit to analyze protein subcellular localization in a large-scale manner.

## Results and Discussion

### Special agroinfiltration method could benefit transient transformation

**Figure 1:**
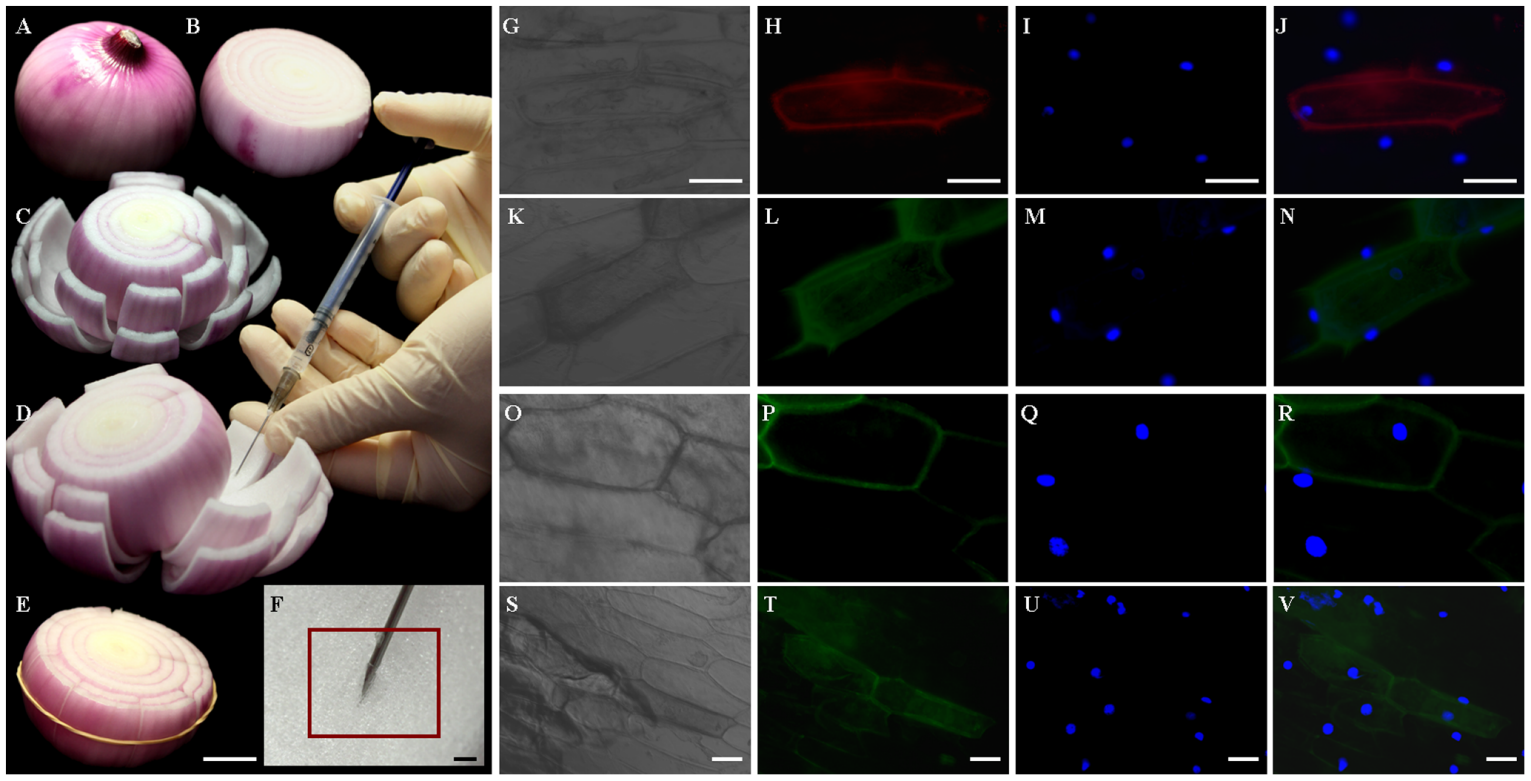
*Agrobacterium* mediated *in planta* transient transoformationin living onion epidermal cells. (A–F) Operational process of the modified agroinfiltration, (A) Onion bulb without outer scales, (B, C) The cut onion bulb prepared for subsequent injection, (D) The injection of *Agrobacteria*, (E) Bind injected cut scales together with elastic for further incubation, scale bar = 2.5 cm. (F) The magnification of injection location, scale bar = 4 mm. (G–V) Onion epidermal cells were transformed with constructs of pCM1205-RFP (G, H, I and J), pLPGM202 (K, L, M and N), pLPGM413 (O, P, Q and R) and pLPGM113 (S, T, U and V). Bright field images (G, K, O and S), UV excited fluorescence images (L, P, T and H), UV excited DAPI staining images (I, M, Q and U) and the merged images of fluorescence and DAPI (J, N, R and V), scale bar = 10 µm.

**Figure 2:**
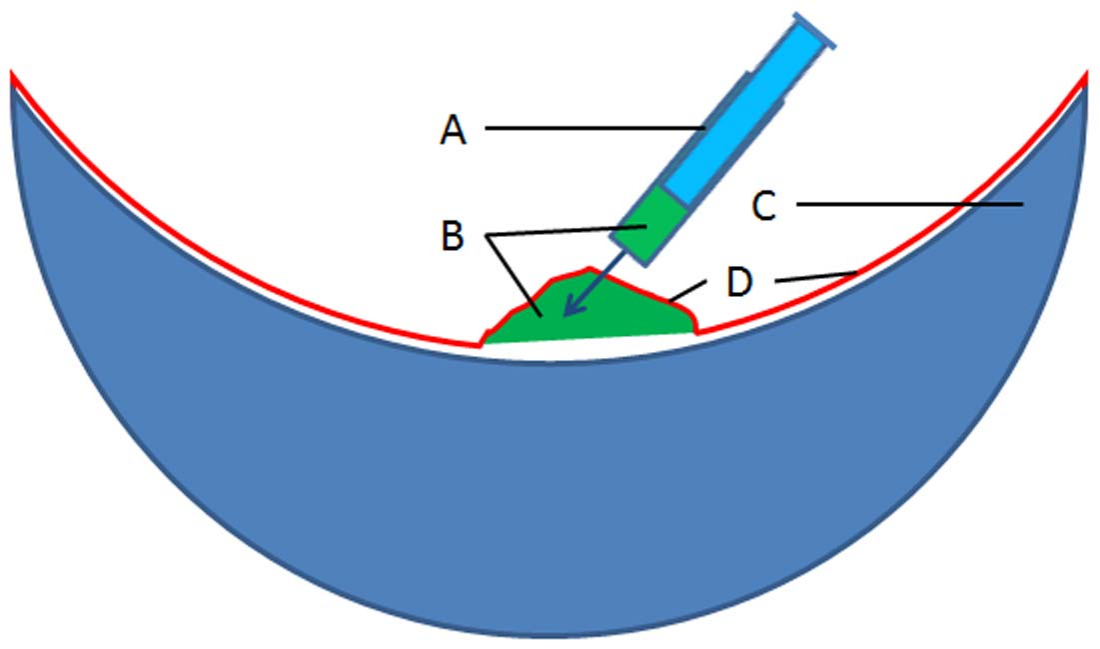
Schematic diagram of forming agroinfiltration bubble by injecting agroinfiltration liquid into the interface between adaxial epidermis and mesophyll of onion bulb scale. (A) Syringe. (B) Agroinfiltration liquid. (C) Mesophyll of onion bulb scale. (D) Adaxial epidermis of onion bulb scale.

We first modified the agroinfiltration method previously used in *Nicotiana tabacum* [36], [37] in order to increase the efficiency of transient transformation. About 200 µl agroinfiltration liquid was slowly injected into the interfaces between adaxial epidermis and mesophyll of onion bulb scales by using a plastic syringe with needle, which resulted in an agroinfiltration bubble at the injection spot filled with infiltration liquid occupying about 1 cm^2^ area of epidermis (Figure 1). The formation of agroinfiltration bubble was demonstrated in the schematic diagram (Figure 2). We found that 1 cm^2^ agroinfiltration area with forming bubble gave higher transformation efficiency than a larger agroinfiltration area without forming bubble. The reason could be that forming agroinfiltration bubble filled with agroinfiltration liquid gave the infiltrated epidermal cells more chance to be infected by agrobacteria because the ratio of agrobacteria to infiltrated epidermal cells was higher. The special injection method could contribute to the high transformation efficiency of the developed transient transformation method. In contrast with other plant material, the adaxial epidermis and mesophyll of onion bulb scales are more flexible to be separated to form an interface bubble, which can take more agroinfiltration liquid.

### Optimization of optical density (OD) of bacteria and agroinfiltration duration

The OD of bacteria and agroinfiltration duration were optimized. It showed that among the different agroinfiltration durations (24 h, 48 h, 72 h and 96 h), three-day agroinfiltration gave the significantly higher transformation efficiency (Table 1). While among three different concentrations of bacteria, agroinfiltration liquid with OD_600_ of 0.10 resulted in significantly higher transformation efficiency (Table 1). Considering both elements, the conditions of agroinfiltration liquid with OD_600_ of 0.10 and three-day infiltration were adopted to achieve the highest transformation efficiency of 43.87% (Table 1).

**Table 1:** Effects of bacterial concentrations and durations of agroinfiltration.

Agroinfiltration durations	Transient transformation efficiencies (%) by using infiltration of *Agrobacterium* with different concentrations		
	0.05 (OD_600_)	0.10 (OD_600_)	0.15 (OD_600_)
24 h	3.30±0.259 cC	12.03±0.282 dD	5.10±0.216 dD
48 h	4.30±0.240 bB	23.77±0.274 cC	12.37±0.256 bB
72 h	13.10±0.326 aA	43.87±0.431 aA	22.53±0.335 aA
96 h	4.77±0.164 bB	17.97±0.282 bB	7.70±0.199 cC

Note: three samples per combination of bacterial concentration and infiltration duration were investigated for ten 2 mm^2^ epidermal areas. Each efficiency value represents the mean of transformation efficiencies of thirty replicates of 2 mm^2^ epidermal areas from three samples, and the standard errors were calculated by using Excel. Different capital and lowercase letters within the same column exhibit significant difference at the 1% and 5% probability level according to the Duncan test of SPSS 10.0 statistic analysis.

### Agroinfiltration liquid with special combination of components contributed to the high efficiency of transient transformation

The components of infiltration liquid and transfection conditions were referenced to relevant reports [13], [38]–[42] and with special modification. Based on agroinfiltration liquid for floral dip stable transformation and other transient transformation assays, the modified agroinfiltration liquid included 6-benzylaminopurine (BAP), Silwet L-77, D-glucose, acetosyringone (AS), magnesium chloride hexahydrate (MgCl_2_), calcium chloride dehydrate (CaCl_2_) and MES-KOH. To our knowledge, adding plant growth regulator BAP into the agroinfiltration liquid for transient transformation was seldom, although it had previously been applied in floral dip stable transformation. As an analogue of cytokinin, BAP plays roles in promoting cell division and prevent cell senescence, adding of low concentration of BAP in the agroinfiltration liquid could benefit to keep onion epidermal cells vigorous during agroinfiltration. Surfactant, glucose, osmotic buffer, calcium and magnesium ions, and acetosyringone were also supplemented to benefit the transient transformation.

In order to confirm the necessary roles of all the components, the transformation efficiencies of different infiltration liquids without one of the components were investigated (Table 2). The results indicated that all the components of agroinfiltration liquid were necessary and significantly contributed to the high efficiency of *Agrobaterium*-mediated transient transformation in onion epidermal cells.

**Table 2:** Effects of different components of agroinfiltration liquid (OD_600_ = 0.10).

Infiltration components	Transient transformation efficiencies (%) of different agroinfiltration durations			
	24 h	48 h	72 h	96 h
Complete components	12.93±0.262 aA	22.97±0.376 aA	43.88±0.330 aA	15.68±0.070 aA
D-glucose-	0.15±0.014 fF	0.91±0.013 gG	2.61±0.040 gG	2.40±0.031 hG
CaCl_2_-	1.21±0.050 eE	2.43±0.047 fF	5.99±0.036 fF	2.55±0.045 gG
MES-KOH-	3.30±0.048 cC	5.24±0.059 dD	13.32±0.089 bB	6.88±0.081 cC
BAP-	2.13±0.062 dD	4.21±0.049 eE	8.28±0.043 dD	5.67±0.043 dD
Silwet L-77-	7.22±0.083 bB	10.07±0.126 bB	12.71±0.082 cC	9.43±0.045 bB
MgCl_2_-	2.42±0.065 dD	6.34±0.040 cC	8.39±0.040 dD	4.32±0.029 eE
AS-	1.15±0.041 eE	4.39±0.044 eE	7.29±0.253 eE	3.42±0.054 fF

Note: three samples per unit of infiltration components were investigated for ten 2 mm^2^ epidermal areas. Each efficiency value represents the mean of transformation efficiencies of thirty replicates of 2 mm^2^ epidermal areas from three samples (the percentage of positive cells in total cells per unit area), and the standard errors were calculated by using Excel. Different capital and lowercase letters within the same column exhibit significant difference at the 1% and 5% probability level according to the Duncan test of SPSS 10.0 statistic analysis. The symbol “-”, represents that the agroinfiltration liquid included all the components except the referred component.

### The developed method is advantageous to particle bombardment and onion is more suitable for the developed method than tobacco and *Arabidopsis*

Through analyzing the side by side experiments of transforming pLPGM413 into onion epidermal cells with the developed method and particle bombardment, it indicated that the developed method resulted in significantly high transformation efficiency and showed advantages on cost and equipment needed (Table 3).

**Table 3:** Comparison of agroinfiltration and particle bombardment methods on transformation in onion epidermis.

Comparison items	Transient transformation methods	
	Agroinfiltration	Particle bombardment
Transformation time	One day (about 12.93% transformation efficiency)	One day (about 4.67% transformation efficiency)
Cost	Low	High
Special equipment	Not needed	Biolistic equipment
Transformation efficiency (%)	43.73±0.23	4.67±0.11

Note: three samples per unit of infiltration components were investigated for ten 2 mm^2^ epidermal areas. The efficiency value represents the mean of transformation efficiencies of thirty replicates of 2 mm^2^ epidermal areas from three samples (the percentage of positive cells in total cells per unit area) in the part of transformation efficiency, and the standard errors were calculated by using Excel.

The side by side experiments of *Agrobacterium* mediated transient transformation in onion, tobacco and *Arabidopsis* [14] with the same agroinfiltration liquid showed that the developed transient transformation method of onion epidermis gave significantly high transformation efficiency than tobacco and *Arabidopsis* (Table 4). The developed method of transformation in onion epidermis took only 5 days from the pretreatment of onion bulb to the best time-point for visualizing FFP signals, which is shorter than those in tobacco and *Arabidopsis* if considering the preparation time of plants [14]. In addition, the developed method is easier to get ideal images because onion epidermis has transparent cells arranged in a monolayer. Therefore, the developed method of onion has advantage to those of tobacco and *Arabidopsis*.

**Table 4:** Comparison of transient transformation efficiencies in different plant materials by using agroinfiltration.

Plant materials	Transient transformation efficiencies (%) by using infiltration of *Agrobacterium* (OD_600_ = 0.10) with different agroinfiltration durations			
	24 h	48 h	72 h	96 h
Arabidopsis(Col-0)	1.31±0.041 cC	2.56±0.066 cC	1.79±0.043 cC	1.63±0.053 cC
Tobacco(Xanthi)	1.93±0.044 bB	4.97±0.051 bB	6.54±0.074 bB	5.22±0.035 bB
Onion(Hongtaiyang)	12.34±0.098 aA	23.38±0.243 aA	43.35±0.343 aA	14.99±0.082 aA

Note: three samples per material were investigated for ten 2 mm^2^ areas of agroinfiltrated epidermal cells. Each efficiency value represents the mean of transformation efficiencies of thirty replicates of 2 mm^2^ epidermal areas from three samples (the percentage of positive cells in total cells per unit area), and the standard errors were calculated by using Excel. Different capital and lowercase letters within the same column exhibit significant difference at the 1% and 5% probability level according to the Duncan test of SPSS 10.0 statistic analysis. For onion two-day pretreatment was conducted before agroinfiltration.

### Pretreatment of onion bulb benefited the *Agrobacterium* mediated transient transformation in onion epidermis

The effects of different pretreatment times of onion bulb before injection of agroinfiltration liquid were evaluated (Table 5). It indicated that with the increase of pretreatment time the transformation efficiencies were increased (Table 5). However, 48-h and 72-h pretreatments resulted in similar transformation efficiencies, which are significantly higher than those resulted from 0-h and 24-h pretreatments (Table 5). It implied that 48-h pretreatment could be the suitable pretreatment time, which helped to achieve the highest transformation efficiency with the least time (Table 5).

**Table 5:** Effects of different pretreatment time of onion before *Agrobacterium* infection.

Pretreatment time	Transient transformation efficiencies (%) by using infiltration of *Agrobacterium* concentration (OD_600_ = 0.10) with different agroinfiltration durations			
	24 h	48 h	72 h	96 h
0 h	2.78±0.052 cC	5.97±0.066 cC	8.74±0.056 dC	5.20±0.048 dD
24 h	8.10±0.068 bB	15.01±0.158 bB	24.10±0.279 cB	13.17±0.272 cC
48 h	12.93±0.203 aA	22.43±0.436 aA	42.40±0.309 bA	15.77±0.298 bB
72 h	13.03±0.286 aA	22.10±0.480 aA	43.57±0.513 aA	17.71±0.276 aA

Note: three repeat samples for each treatment were investigated by observing ten 2 mm^2^ epidermal areas of each sample. Each efficiency value represents the mean of transformation efficiencies of thirty replicates of 2 mm^2^ epidermal areas from three samples (the percentage of positive cells in total cells per unit area), and the standard errors were calculated by using Excel. Different capital and lowercase letters within the same column exhibit significant difference at the 1% and 5% probability level according to the Duncan test of SPSS 10.0 statistic analysis.

### Monitoring of transformation and subcellular localization confirmation

The process of transient transformation was monitored by using four different plasmids carrying report genes mediated by *A*. *tumefaciens* strain GV3101, which constructed as the following expression binary vectors, pLPGM413 carrying *RcSERK1* (*somatic embryogenesis receptor-like kinase 1* of *Rosa canina*)[43] and *GFP* (green fluorescent proteins), pLPGM413 modified from pSAT6-GFP-N1 [44]–[47] by adding T-DNA border region of pCAMBIA1303, pLPGM113 modified from pEZS-NL-GFP [48] by adding T-DNA border region of pCAMBIA1303 and pCM1205-RFP (red fluorescent protein) carrying *RFP* [49], [50]. The *Agrobacterium* cells harboring above vectors were grown in liquid yeast extract and beef extract medium (YEB) until stationary phase and then re-suspended in infiltration liquid with above special components. Two to three days after injection of infiltration liquid, obvious GFP/RFP signals corresponding to different report genes could be observed in epidermal cells (Figure 1). It indicated that the developed agroinfiltration method can efficiently mediate *in vivo* transient expression of genes in living onion epidermal cells. For pCM1205-RFP, pLPGM202 and pLPGM113, the proteins encoded by their harboring genes were localized throughout the cells (Figure 1), and the fusion protein encoded by *RcSERK1-GFP* in pLPGM413 was localized in cell membrane (Figure 1). The results were the same as those of previous studies [43]–[50], suggesting the authenticity of the developed method.

The fluorescence signals of the nuclei in the cells transformed by pCM1205-RFP, pLPGM202 and pLPGM113 overlapped with those nuclei stained by 4′,6-diamidino-2-phenylindole (DAPI) (Figure 1), while no fluorescence signals were observed in nuclei of the cells transformed by pLPGM413. It further confirmed the credibility of the developed transient transformation method. It indicated that the developed transient transformation method in onion epidermal cells could be used to determine subcellular localization of the target proteins with the consistence to other transient transformation systems.

### Conclusions

We have developed a rapid and low-cost transient transformation assay by using *in-planta* agroinfiltratoin in living onion epidermal cells, which transformation efficiency is higher than those of the conventional transient assays. It demonstrated that subcellular localization of proteins evaluated by using this method was same as that by other transient transformation assays and DAPI staining. Since it is rapid, efficient and low-cost, this method is suitable for large-scale analyses of protein subcelluar localization.

## Materials and Methods

### Plant material

Cultivated onion “Hongtaiyang” (*A. cepa* L.), which is a kind of Chinese onion cultivar with red skin, was used as experimental material. Before the injection of agroinfiltration liquid the onion bulbs pre-grew in darkness at 28°C for different days to evaluate the effect of different pretreatment time of onion bulb (Table 5). After injection the onion bulbs were incubated at 28°C in darkness for the agroinfiltration.

Tobacco (*Nicotiana tabacum* L. var. Xanthi nc.) was grown under 25°C with photoperiod of 16 h light (200 µmol m^−2^ s^−1^) and 8 h darkness. The leaves of fifty-day old plants were injected with agroinfiltration liquid from abaxial surface with syringe.

*A. thaliana* (Colombia ecotype) was grown under 20°C with photoperiod of 16 h light (120 µmol m^−2^s^−1^) and 8 h darkness. The leaves of 15 days old plants, the injection method was same as that of tobacco.

### GFP/RFP-based constructs

The binary expression vector pLPGM202 harboring *GFP* genes was modified from pSAT6-GFP-N1 provided by Prof. Tao Wang (State Key Laboratory of Agro-biotechnology, China Agricultural University) by adding T-DNA border region of pCAMBIA1303, which contains the right border, kanamycin (Kan) resistance gene for bacterial resistance selection and the left border. The steps of creating pLPGM202 was as follows: the T-DNA border region (2888-9172) of pCAMBIA1303 was amplified by using PrimeSTAR® GXL DNA Polymerase (TaKaRa, Japan) with PCR primers of Fsat-YR (*Not*I-GTAAACCTAAGAGAAAAGAG) and Rsat-YR (*PI*-*Psp*I-TTTGCCTGTTTACACCACAAT) carrying the restriction enzyme cutting sites of *Not*I and *PI*-*Psp*I, respectively. The PCR products were ligated into pSIMPLE-19 *Eco*RV/BAP vector by T4 DNA Ligase (TaKaRa, Japan) and sequenced to confirm the success of ligation. The constructed recombinant pSIMPLE-19 *Eco*RV/BAP with the T-DNA border region of pCAMBIA1303 was transformed into *Escherichia coli* DH5α for propagation. The pSAT6-GFP-N1 were digested with partial digestion method by using *Not*I and *PI*-*Psp*I (NEB, USA), the target digested pSAT6-GFP-N1 was obtained with electrophoresis method. The T-DNA border region of pCAMBIA1303 was obtained from the recombinant pSIMPLE-19 *Eco*RV/BAP through complete digestion with *Not*I and *PI*-*Psp*I, and cloned into the target partially-digested pSAT6-GFP-N1 to form pLPGM202 (Figure S1). The successful construction of pLPGM202 was confirmed by sequencing (Sangon Biotech, China).

The binary expression vector pLPGM113 was modified from pEZS-NL-GFP provided by Dr. Liang Zhang (Department of Life Science, Henan Normal University) by adding T-DNA border region (2888-9172) of pCAMBIA1303 same as that for pLPGM202 described above. The steps of creating pLPGM113 were similar as those of pLPGM202 to be simply described as follows: The T-DNA border region of pCAMBIA1303 was amplified with the primers Fnl-YR (*Pst*I-GTAAACCTAAGAGAAAAGAG) and Rnl-YR (*Spe*I-TGTTTACACCACAATATATCC) carrying the restriction enzyme cutting sites of *Pst*I and *Spe*I, respectively. The PCR products were ligated into pSIMPLE-19 *Eco*RV/BAP vector by T4 DNA Ligase (TaKaRa, Japan) and propagated in *E. coli* DH5α. The pEZS-NL-GFP were digested with partial digestion method by using *Pst*I and *Spe*I (NEB, USA), the target digested pEZS-NL-GFP was obtained with electrophoresis method. The T-DNA border region of pCAMBIA1303 was obtained from the recombinant pSIMPLE-19 *Eco*RV/BAP through complete digestion with *Pst*I and *Spe*I, and cloned into the target partially-digested pEZS-NL-GFP to form pLPGM113 (Figure S2). The successful construction of pLPGM113 was confirmed by sequencing (Sangon Biotech, China).

The binary expression vector pCM1205-RFP harboring *RFP* gene was provided by Dr. Wencai Qi (Bioengineering Department, Zhengzhou University).

The cDNA of *RcSERK1* was isolated from cDNA library of *R*. *canian* PLBs and its sequence was deposited in GenBank as accession number of HM802242. Coding sequence of this gene (without stop codon) was amplified by PCR using Platinum *pfx* DNA polymerase (Invitrogen, USA) with the following primers: *RcSERK1* (F-SKs, forward: 5′-CCCAAGCCTCATGGATAGCAGGCTT-3′; R-SKs, reverse: 5′-TCCCCCGGGCCTTGGACCAGATAAC-3′). The PCR products were digested with *Hind*III and *Sma*I, subsequently ligated into the *Hind*III/*Sma*I sites between the CaMV 35S promoter and *GFP* of pLPGM202 to construct the expression binary vector pLPGM413.

### *Agrobacterium* infiltration

The vectors, pCM1205-RFP, pLPGM113, pLPGM202 and pLPGM413, were transformed into *A*. *tumefaciens* strain GV3101 for further transient transformation in onion epidermis. Positive *Agrobacterium* harboring pCM1205-RFP was selected and cultivated in YEB media supplemented with 100 mg/L rifampicin and 25 mg/L chloramphenicol. Positive *Agrobacterium* harboring pLPGM113, pLPGM202 and pLPGM413 were selected and cultivated in YEB media supplemented with 100 mg/L rifampicin and 100 mg/L Kan. Positive *Agrobacterium* cultivated overnight at 28°C were harvested at OD_600_ of 1.5 to 2.0, centrifuged at 5000 rpm for 10 min and re-suspended in 50 ml of infiltration liquid, and the centrifugation and resuspension procedure was repeated three to five times. Finally, *Agrobacterium* cells were diluted in agroinfiltration liquid to appropriate concentration for agroinfiltration. Different agroinfiltration durations (24 h, 48 h, 72 h and 96 h) and *Agrobacterium* concentrations (OD_600_ 0.05, 0.10 and 0.15) were evaluated to determine conditions to obtain high transformation efficiency (Table 1).

The complete infiltration liquid was made as following: 41.65 mM D-glucose, 100 mM CaCl_2_, 100 mM MES-KOH (pH 5.6) stock solution, 0.011 µM BAP, 0.01% Silwet L-77, 0.05 mM MgCl_2_ and 12.5 mM AS (made with DMF, dimethylformamide) stock solution, and suitable amount of ddH_2_O to make final volume to 20 ml. About 200 µl infiltration liquid with *Agrobacterium* carrying constructed vectors were injected into the interface of adaxial epidermis and mesophyll of onion scales to make a bubble for agroinfiltration. In order to investigate whether all the components are necessary, different infiltration liquid without one of the components were used to evaluated the effect of different components of the infiltration liquid (Table 2).

### Particle bombardment

The adaxial epidermis was obtained from onion bulb and placed on MS medium for 1-day incubation. The binary expression vector pLPGM413 was transformed into the onion epidermal cells with particle bombardment method as described by [51].

### DAPI staining

To visualize nuclei, the epidermis was stained with DAPI (5 µg/mL, sigma, USA). Materials were soaked in the dye liquid phosphate buffer solution (PBS) (pH 7.0; DAPI: PBS (v/v) = 1∶1000) and kept in darkness for 20 min. Pieces of onion epidermis were arranged on slides to make wet mounts, the made slides were observed and photographed in dark-field of fluorescence microscope (Olympus BX 61, Japan).

### Microscopic investigation

Transformation efficiency was determined by calculating the proportion of positive epidermal cells with fluorescent signals among the cells in 2 mm^2^ epidermis area that was measured with micro ruler under microscope. Images of epidermal cells were taken by using a motorized fluorescence microscope with a mirror unit (U-MNU2), dichroic mirror (DM400), excitation filter (BP360) and barrier filter (BA420) for DAPI (DNA staining). Images of epidermal cells positive for GFP were taken with a mirror unit (U-MSWB2), dichroic mirror (DM500), excitation filter (BP470-490) and barrier filter (BA520IP) for GFP. Images of epidermal cells positive for RFP were taken with a mirror unit (U-MSWG2), dichroic mirror (DM570), excitation filter (BP530-550) and barrier filter (BA590).

### Statistical analysis

All statistical analyses were performed with SPSS (version 10.0).

## Supporting Information

Figure S1 **The sketch map of pLPGM202 originated from pCAMBIA1303 and pSAT6-GFP-N1.** Details of creating the vector are given in the Materials and methods.
(TIF)

Figure S2 **The sketch map of pLPGM113 originated from pCAMBIA1303 and pEZS-NL-GFP.** Details of creating the vector are given in the Materials and methods.
(TIF)
