# Supplementary figures and images for: A Rapid, Highly Efficient and Economical Method of Agrobacterium-Mediated In planta Transient Transformation in Living Onion Epidermis
Source: PLoS One. 2014 Jan 8;9(1):e83556. doi: 10.1371/journal.pone.0083556 (PMC3885512; doi:10.1371/journal.pone.0083556)

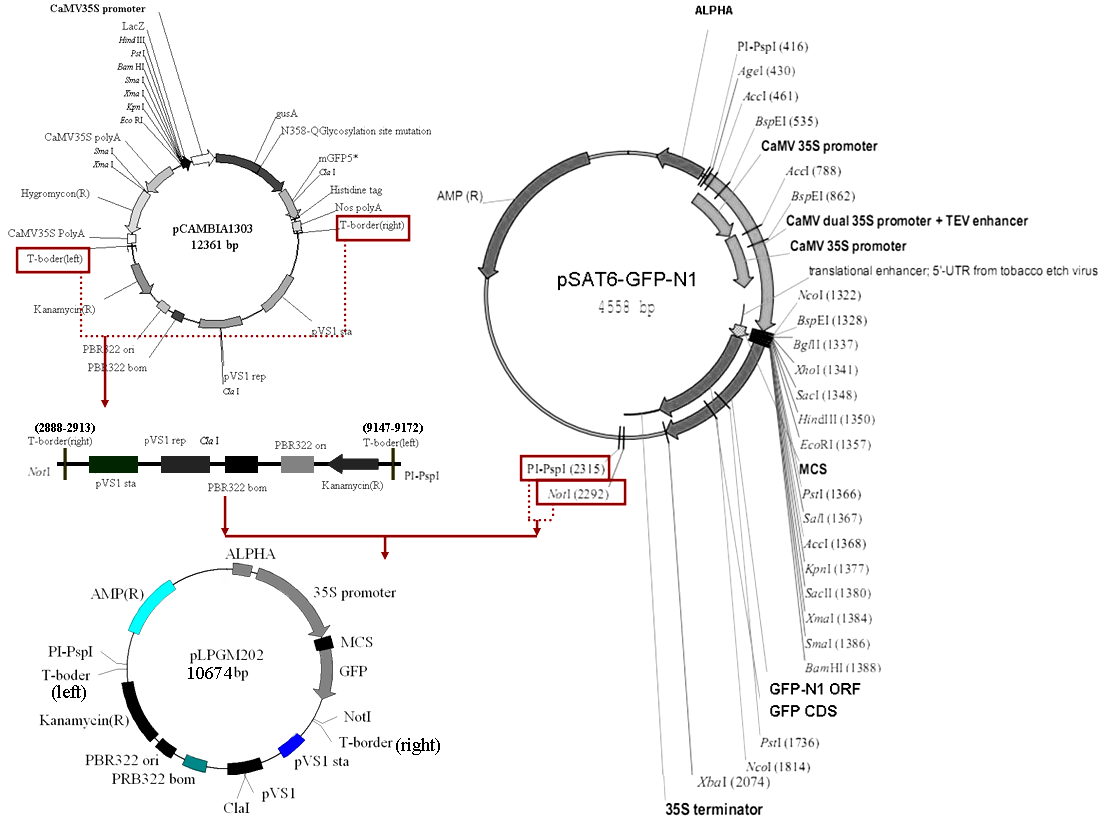

Supplement: Figure S1 — The sketch map of pLPGM202 originated from pCAMBIA1303 and pSAT6-GFP-N1. Details of creating the vector are given in the Materials and methods. (TIF) [file pone.0083556.s001.tif]

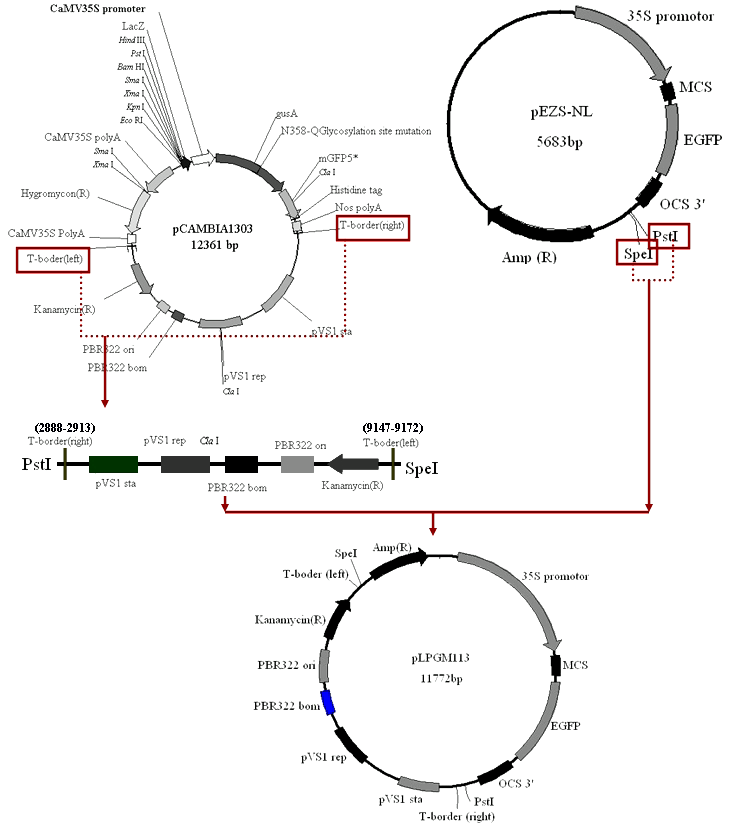

Supplement: Figure S2 — The sketch map of pLPGM113 originated from pCAMBIA1303 and pEZS-NL-GFP. Details of creating the vector are given in the Materials and methods. (TIF) [file pone.0083556.s002.tif]
